# A Criterion of Colorectal Cancer Diagnosis Using Exosome Fluorescence-Lifetime Imaging

**DOI:** 10.3390/diagnostics12081792

**Published:** 2022-07-24

**Authors:** Alexey V. Borisov, Olga A. Zakharova, Alisa A. Samarinova, Natalia V. Yunusova, Olga V. Cheremisina, Yury V. Kistenev

**Affiliations:** 1 Laboratory of Laser Molecular Imaging and Machine Learning, National Research Tomsk State University, 634050 Tomsk, Russia; borisov@phys.tsu.ru (A.V.B.); za.olga.90@yandex.ru (O.A.Z.); alisasamarinova@gmail.com (A.A.S.); 2 Laboratory of Tumor Biochemistry, Cancer Research Institute, Tomsk National Research Medical Center RAS, 634009 Tomsk, Russia; bochkarevanv@oncology.tomsk.ru (N.V.Y.); cheremisinaov@oncology.tomsk.ru (O.V.C.)

**Keywords:** colorectal cancer, exosomes, two-photon exited autofluorescence, fluorescence-lifetime imaging microscopy

## Abstract

This study was aimed to investigate the applicability of the exosome fluorescence-lifetime imaging microscopy (FLIM) for colorectal cancer (CRC) diagnosis. Differential ultra-centrifugation was used to extract exosomes from the blood plasma of 11 patients with colon polyps (CPs) and 13 patients with CRC at the T2-4, N0-3, and M0-1 stages. Analysis was performed using a two-photon FLIM device. In total, 165 and 195 FLIM images were recorded for the CP and CCR patient groups, respectively. Two classes of exosomes differentiated by autofluorescence average lifetime $t_{m}\;$were discovered in the samples. The first class of exosomes with $t_{m}$ = (0.21 ± 0.06) ns was mostly found in samples from CRC patients. The second class with $t_{m}$ = (0.43 ± 0.19) ns was mostly found in samples from CP patients. The relative number of “CRC-associated” exosomes $N_{ch}\;$in the FLIM dataset was shown to be very small for the CP patient group and large for the CRC patient group. This difference was statistically significant. Therefore, the suggested CRS diagnostics criterion can be as follows. If $N_{ch}$ > 0.5, the probability of CRC is high. If $N_{ch}$ < 0.3, the probability of CRC is low.

## 1. Introduction

According to the World Health Organization, colorectal cancer (CRC) is the third most common type of cancer and the second leading cause of cancer-associated deaths among men and women worldwide [1,2]. Usually, four stages of CRC are considered, depending on the five-year survival rate (from 90% to less than 1%). CRC is asymptomatic in the early stages and involves the rapid formation of metastases in the late stages [2,3,4]. Early diagnosis can save up to 90% of CRC patients [4,5,6].

- In clinical practice, the following methods of CRC diagnostics have become the most widespread [7,8,9]:
- Finger rectal examination;
- Irrigoscopy (X-ray examination of the intestine);
- Test for hidden blood in feces;
- Capsule endoscopy (evaluation using an endocapsule with a digital video camera that passes through all parts of the intestine);
- Colonoscopy (endoscopic diagnostic method using a colonoscope with a video camera, if necessary with a biopsy);
- Cancer markers’ blood tests.

With the exception of blood tests, similar diagnostic tools do not provide early CRC detection. As an example, in Russia, the diagnosis of CRC is made at stage IV in 27.7% of cases, at stage III in 23.9% of cases, at stage II in 37.5% of cases, and at stage I in 8.8% of cases. Blood tests are based on checking for the products of varied cell metabolism that can potentially catch cancer at the early stages. Currently, more than two hundred types of specific molecular compounds have been identified, but only about 30 of them are considered to be potential CRC biomarkers [10]. The main marker of rectal cancer is carcinoembryonic antigen [11]. However, the latter is characteristic not only for the malignant process but also some inflammatory, autoimmune and other benign diseases of the internal organs. Therefore, the discovery of more specific CRC blood biomarkers is vital.

Exosomes are small (30–100 nm) extracellular vesicles released by cells to provide intercellular communication. Exosomes are stable for a long time, are carried by the blood and lymph flow, and can be detected in the body away from the location of the cells secreting them [12,13,14]. The ExoCarta database contains a library of lipids, proteins, more than 1600 mRNAs, and more than 760 miRNAs detected in exosomes [15]. The biochemical cargo of an exosome depends on its origin. This is the reason that cancer-associated exosomes stimulate tumor growth, angiogenesis, extracellular matrix remodeling, metastasis, and immune surveillance. Exosomes are considered to be a potential biomarker of cancer [16,17], including colorectal cancer [18,19].

The most commonly used methods of exosome study are as follows. Flow cytometry makes it possible to detect extracellular vesicles and surface antigens associated with their origin, thus allowing for subpopulation identification. The main limitations are sensitivity and spatial resolution. Typical flow cytometers can detect vesicles larger than 200 nm; newer models can detect particles from larger than 150 nm in size [20]. At high concentrations, several exosomes can be identified as one by flow cytometry. Dynamic light scattering provides information about an exosome’s size in an interval from 1 nm to 6 μm. However, this method only provides reliable data when one type of exosome is present in the suspension and is less accurate for polydisperse suspensions [21]. Nanoparticle tracking analysis determines the size and concentration of particles by analyzing their trajectory in suspension, though its disadvantages include an inaccuracy of particle size determination, a lack of selectivity to structure, and aggregation [21,22]. Transmission electron microscopy has a spatial resolution of about 1 nm [21]. Its main disadvantages are working only with thin samples (not more than 100 nm) and a risk of sample damage. Atomic force microscopy provides a three-dimensional topography of an object’s surface with very high spatial resolution. Its disadvantage is the presence of artifacts affecting the probe from the surface during the interaction of the tip with a sample [21]. IR spectroscopy makes it possible to determine the chemical profile of an object [20], but it is not applicable to a sample containing water, since this solvent strongly absorbs IR radiation. Raman spectroscopy can differentiate exosomes depending on the lipid/protein content in the membrane along with other various surface modifications. The main disadvantage of Raman spectroscopy is the significantly smaller cross-section of Raman scattering compared to that of resonance absorption [21]. 

Modern optical coherence tomography (OCT), laser confocal microscopy, and multiphoton microscopy have a spatial resolution of about 1–2 μm [23,24,25]. Multicolor two-photon excited autofluorescence microscopy, two-photon exited autofluorescence (TPAF) microscopy, and fluorescence lifetime imaging microscopy (FLIM) make it possible to visualize a single nanoobject in the case of its bright emission in a spectral range placed far from a wavelength of pumping femtosecond laser [25,26,27].

The aim of this paper was to develop an approach in CRC diagnostics using the exosomes analysis by TPAF.

## 2. Materials and Methods

Blood samples from 11 patients with colon polyps (CPs) (54.9 ± 1.6 years) and 13 CRC patients with the T2-4, N0-3, and M0-1 stages (58.6 ± 1.6 years) were collected in the Cancer Research Institute of Tomsk National Research Medical Center (Tomsk, Russia). The CRC group comprised patients with colon cancer and upper-rectal cancer. Exclusion criteria for the CRC group were: multiple primary CRC, mid-rectal cancers, and low-rectal cancers. Patients with CPs were considered a control group. Colon polyps have a high risk of malignancy and are considered a precancerous stage but still not a cancer. Therefore, it is important to distinguish between these states to provide an adequate therapy.

The study was conducted in accordance with the Declaration of Helsinki, and the protocol was approved by the Local Ethics Committee of the Cancer Research Institute of Tomsk National Research Medical Center (Tomsk, Russia) (protocol No.2, 15 January 2020). All participants were informed of the purpose and nature of the treatment and gave their informed consent. All patients with Stage II and III CRC underwent radical surgery (hemicolectomy or colon resection). The stage of the disease was established after surgery in accordance with the international TNM Classification of Malignant Tumors (8th Edition).

Standard vacuum K3 tubes with an ethylene-diamine-tetraacetate anticoagulant were used to collect peripheral blood from the ulnar vein. Exosomes from blood plasma were extracted by ultrafiltration followed by ultracentrifugation according to the following protocol [17,28]. Shaped elements of the 9 mL volume blood samples were deposited by centrifugation for 20 min at 290 g (bucket rotor) and 4 °C. The supernatant was re-centrifuged for 20 min at 1200 g (bucket rotor) and 4 °C. To remove cellular debris, plasma samples were centrifuged for 20 min at 17000 g (angular rotor) and 4 °C. To remove vesicles over 100 nm, the supernatant was diluted 5 times with a phosphate-salt buffer (PSB) (10 mM phosphate buffer, 0.15 M NaCl, pH 7.5) and filtered through a filter with a pore diameter of 100 nm (Minisart high flow, 16553-K, “Sartorius”, Gottingen, Germany). Exosomes were deposited by ultracentrifugation for 90 min at 100,000 g (bucket rotor) and 4 °C. The precipitate was resuspended in 10 mL of PSB and ultracentrifuged twice under the same conditions. Finally, exosomes were resuspended in 200 µL of PSB, frozen in liquid nitrogen and stored at −80 °C [29]. This protocol had been used for the extraction of various exosome types and, according to the results of nano-tracking analysis, provided the following exosome concentrations in term of median and range values × 10^7^/mL: [16 (8; 20)] for healthy volunteers, [24 (20; 138)] for benign breast diseases patients, [21 (10; 180)] for breast cancer patients, and [22 (13; 154)] for ovarian cancer patients [28]. The variation was not very high. Therefore, these values could be used as a estimation of exosomes concentration in our case.

The samples were analyzed in the Biophotonics Laboratory of Tomsk State University. Before transportation, the sample storage temperature was slowly increased from −80 °C to −20 °C to avoid sticking [29]. The samples were delivered in a special thermal box containing two exosome samples surrounded by two packages of a Green Glade cold storage battery of a 750 mL volume. The temperature in the container was −20 °C. After the container was opened, the exosome samples were placed on ice and allowed to thaw at a 0 °C temperature.

Immediately before the measurements, an exosome sample was stirred using an OPn-8 centrifuge (Dastan, Kyrgyzstan) for 5 min at 1000 g and a temperature of 4 °C. Exosomes were analyzed using a two-photon MPTflex microscope (Jenlab GmbH, Jena, Germany), which has a spatial resolution of about 0.5 microns (horizontal) and 2 microns (vertical). We used a lens with a 1.3 numerical aperture and a 40× magnification. The pump laser pulse duration was about 200 fs, the repetition rate was 80 MHz, and the wavelength was 760 nm. The detector has a spectral filter in the range of 406–660 nm, suitable for TPAF signal registration. The MPTflex microscope has an option for TPAF registration in an FLIM mode.

In order to avoid the photobleaching of exosomes, the laser power was limited to 5 mW according to the melanin vivo visualization protocol [30].

**Figure 1:**
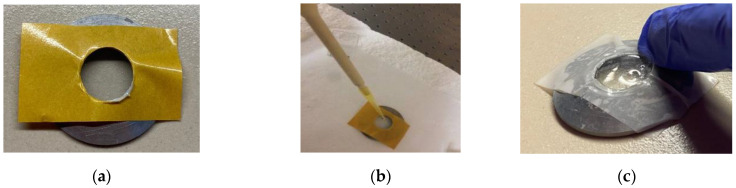
An illustration of an exosome sample process prepared for MPTflex microscope analysis: photographs of a glass plate with adhesive tape (**a**), the process of the exosome sample’s positioning on the glass plate (**b**), and this construction being covered from above with another glass plate (**c**).

A glass plate with a thickness of 100–170 μm was placed on a metal ring. This glass was covered by an adhesive tape about 7 μm thick with a cutout in the form of a round hole (Figure 1a). Next, an exosome sample was applied to this glass in the hole area (Figure 1b). Then, an analogous glass plate covered this construction from above (Figure 1c).

For each sample, FLIM images from depths of about 3–4 μm with sizes of 18 × 18 μm, 52 × 52 μm, and 118 × 118 μm were recorded in five randomly selected spatial positions. Therefore, in total, 165 and 195 FLIM images were recorded for CP and CRC patients, respectively. The FLIM signal $I\left(t\right)\;$registered by a pixel (or group of pixels) of a matrix detector is usually approximated by a multi-exponential decomposition as follows:

(1) $$I\left(t\right)=I\left(0\right)\sum_{i=1}^{N}\alpha_{i}e^{-t/\tau_{i}},$$

where $\alpha_{i}$ and $\tau_{i}$ are the amplitudes and lifetimes of the TPAF signal components, respectively, and $I\left(0\right)$ is the intensity of the measured fluorescence signal at an initial moment. As a rule, analysis is limited by two members of the series [31,32]:

(2) $$I\left(t\right)=a_{1}e^{-t/\tau_{1}}+a_{2}e^{-t/\tau_{2}},\;$$

Average fluorescence lifetime $\tau_{m}$ is often used for TPAF signal characterization:

(3) $$\tau_{m}=\frac{{\;\tau }_{1}a_{1}+{\;\tau }_{2}a_{2}\;}{a_{1}+{\;a}_{2}},\;$$

A phasor-plot approach for FLIM data description and analysis was also used [33,34]. The FLIM data projection on a phasor plane is carried out by the Fourier transform of the FLIM signal $I\left(t\right)\;$:

(4) $$T\left(\omega \right)=\int_{-\infty }^{\infty }I\left(t\right)e^{-j\omega t}dt=\int_{-\infty }^{\infty }I\left(t\right)\cos\left(\omega t\right)dt-j\int_{-\infty }^{\infty }I\left(t\right)\sin\left(\omega t\right)dt,$$

where ω is a frequency. Let us introduce functions $g\left(\omega \right)$ and s$\left(\omega \right)$, characterizing the real and imaginary parts of $T\left(\omega \right):$

(5) $$g\left(\omega \right)=\frac{\int_{0}^{\infty }I\left(t\right)\cos\left(\omega t\right)dt}{\int_{0}^{\infty }I\left(t\right)dt},\;s\left(\omega \right)=\frac{\int_{0}^{\infty }I\left(t\right)\sin\left(\omega t\right)dt}{\int_{0}^{\infty }I\left(t\right)dt}.$$

A suitable presentation of the FLIM signal $I\left(t\right)\;$was shown to be achieved by using $g\left(\omega \right)$ and s$\left(\omega \right)$ at the frequency $\omega_{0}$, where $\omega_{0}\;$is the laser pulses’ repetition angular frequency [35]. The resulting complex value $g\left(\omega_{0}\right)\;-\;$j · s$\left(\omega_{0}\right)\;$is presented on a two-dimensional graph, where the ordinate represents the imaginary part and the abscissa represents the real one. In this approach, an FLIM curve is assigned a point on the phasor plot.

Statistical analysis was performed using the nonparametric Mann–Whitney U tests [36].

## 3. Results

**Figure 2:**
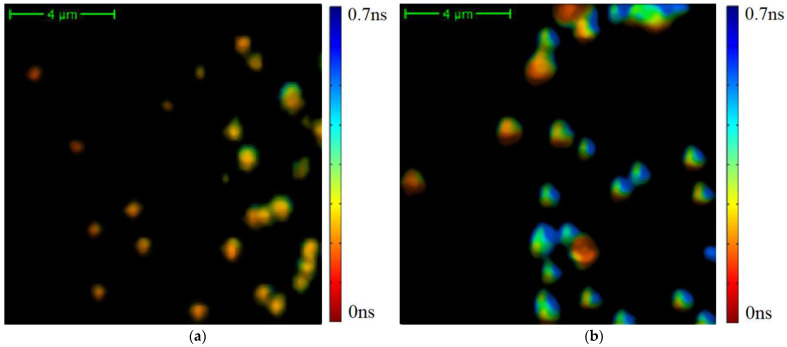
Examples of average TPAF lifetime $t_{m}\;$images of an exosome sample registered by MPTflex microscope for randomly selected CRC (**a**) and CP patients (**b**). The color characterizes $t_{m}$ values according to the presented legend.

Examples of the average TPAF lifetime $t_{m}\;$image for randomly selected CP and CRC patients are shown in Figure 2.

**Figure 3:**
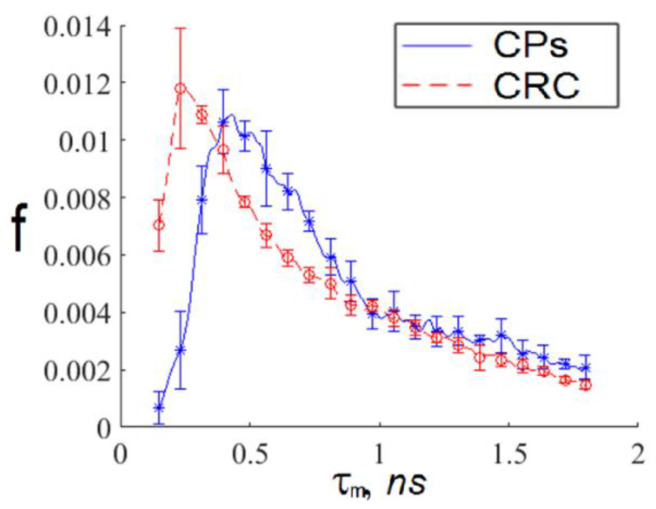
The average TPAF lifetime $t_{m}$ distributions, normalized on an area under the curve, for images of the exosome samples presented in Figure 2. Notations on the legend: CPs—the exosome sample from the randomly selected CRC patient; CRC—the exosome sample from the randomly selected CP patient. The $t_{m}\;$ distribution mean values and standard deviations were calculated by $t_{m}$ value-averaging over images presented in Figure 2a,b.

The TPAF lifetime distribution functions for images presented in Figure 2 are shown in Figure 3.

**Figure 4:**
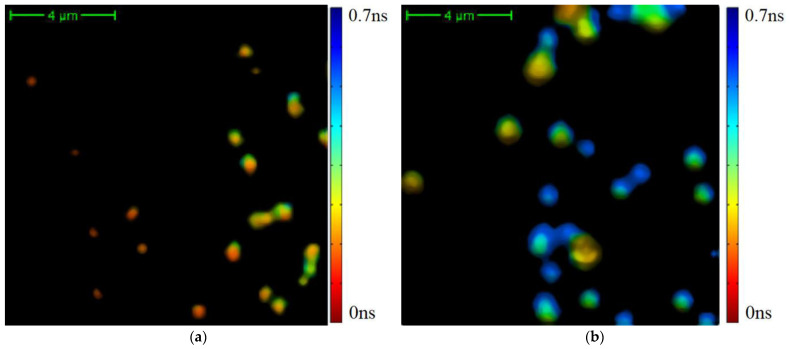
The results of the average TPAF lifetime $t_{m}$ image of exosome samples processed by a cutting filter: the image for the CRC patient (**a**) and the image the CP patient (**b**). The cutting filter had a threshold of 800 photons per pixel. The corresponding unprocessed images are presented in Figure 2.

The small values of the distribution functions presented in Figure 3 correspond to spatial areas that emitted a small number of photons. To focus on spatial areas with large values of emitted TPAF photons and which definitely corresponded to exosomes, we applied a cutting filter with a threshold of 800 photons per pixel (see Figure 4).

**Figure 5:**
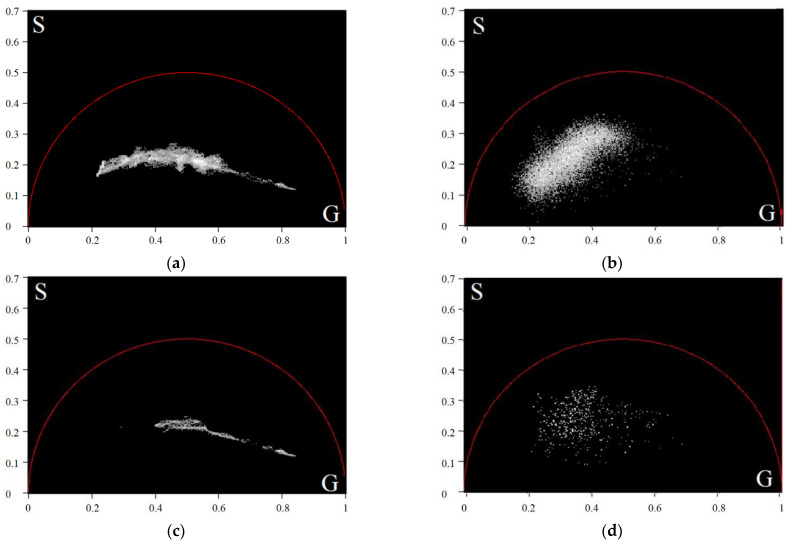
The phasor plots of the average TPAF lifetime $t_{m}\;$images of the exosome samples for a CRC patient before (**a**) and after (**b**) the cutting filter processing; (**c**–**d**)—The same for a CP patient. The cutting filter had a threshold of 800 photons per pixel.

The results of the cutting filter application presented in a phasor plane are shown in Figure 5.

We see that phasor-plot data points for the CRC patient form two spatially separated areas and that there is only one analogous area for the CP patient. To quantitatively describe these areas, the k-means clustering method [37] was applied to the image shown in Figure 5c. The number of classes equal to two was used as an input parameter. To estimate the radii of classes, we used the average value of the Euclidean distance from each point of the set to the center plus its tripled standard deviation (the 3σ rule):

$$R_{j}=\frac{1}{N_{j}}\sum_{i=1}^{N_{j}}r_{i,j}+3\sqrt{\frac{1}{N_{j}}\sum_{i=1}^{N_{j}}{\left(r_{i,j}-\frac{1}{N_{j}}\sum_{i=1}^{N_{j}}r_{i,j}\right)}^{2}}$$

where $r_{i,j}=\sqrt{{\left(g_{c,j}-g_{i,j}\right)}^{2}+{\left(s_{c,j}-s_{i,j}\right)}^{2}}$, $g_{i,j}$ and $s_{i,j}$ are coordinates of a data point in the j−th class, and $g_{c,j},\;s_{c,j},\;\mathrm{and}\;N_{j}$ are coordinates of the center and number of data points in j−th class. The class parameters are:

(6) $$g_{c,1}=0.80,\;s_{c,1}=0.14,\;R_{1}=0.51,\;$$

(7) $$g_{c,2}=0.53,\;s_{c,2}=\;0.23,\;R_{2}=0.71.$$

**Figure 6:**
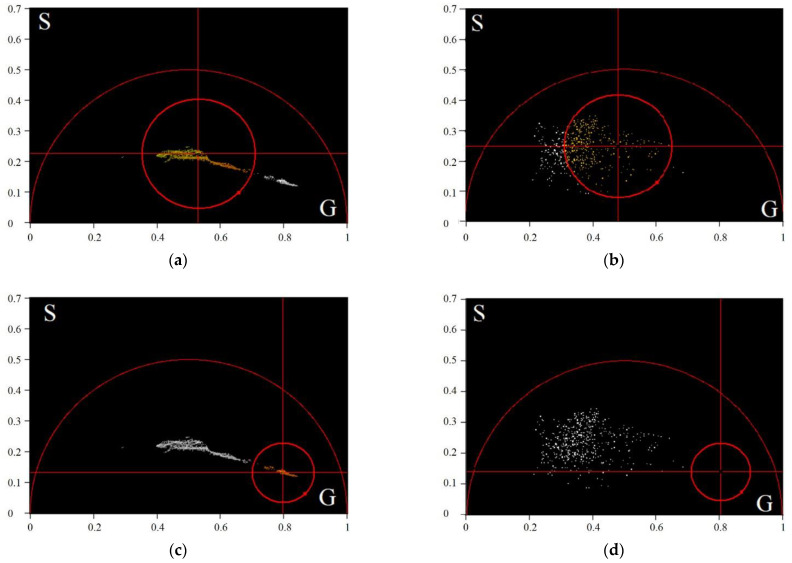
The class areas on phasor plots of the average TPAF lifetime $t_{m}\;$images of the exosome samples presented in Figure 5: the first class position on the phasor plot for the CRC patient (**a**) and the CP patient (**b**), the second class position on the phasor plot for the CRC patient (**c**) and the CP patient (**d**). The first class parameters: $g_{c,1}=$ 0.80, $s_{c,1}=$ 0.14, and $R_{1}=$ 0.51; the second class parameters: $g_{c,2}=$ 0.53, $s_{c,2}=$ 0.23, and $R_{2}=$ 0.71. Here, $g_{c,j},\;s_{c,j},\;\mathrm{and}\;R_{j}$ are coordinates of the center and radius of the j−th class.

The class areas on the phasor plots presented in Figure 5 are shown in Figure 6.

**Figure 7:**
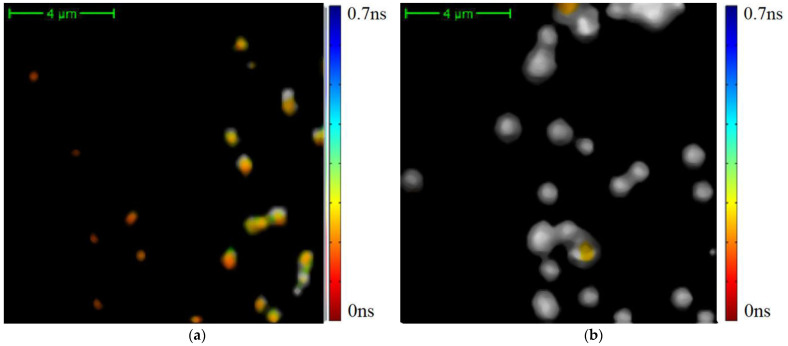
The differentiation of exosomes on the classes described by Formulas (6) and (7). Here, color data points correspond to the exosomes with short average TPAF lifetimes and gray data points correspond to the exosomes with long average TPAF lifetimes. These images are presented unprocessed in Figure 4.

The differentiation of exosomes presented in Figure 4 on the classes is shown in Figure 7.

**Figure 8:**
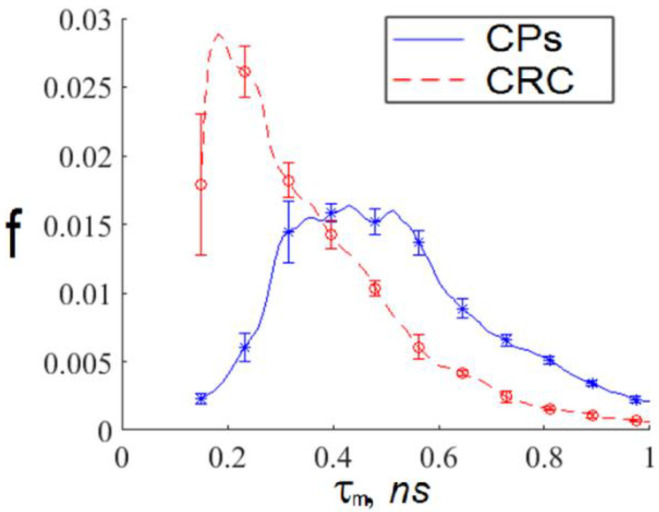
The average TPAF lifetime $t_{m}$ distributions for of the exosome samples, normalized on an area under the curve, for the whole dataset processed by the cutting filter with a threshold of 800 photons per pixel and the circle masks described by Formulas (6) and (7) in a phasor plane. Notations on the legend: CPs—the exosome samples from the whole CRC patient group; CRC—the exosome samples from the whole CP patient group. The $t_{m}\;$ distribution mean values and standard deviations were calculated by $t_{m}$ value-averaging over images presented in Figure 7a,b.

We see that exosomes with short TPAF lifetimes are practically absent on the FLIM image for the CP patient, whereas the opposite situation is true for exosomes with long TPAF lifetimes. Therefore, the class parameters described by Formulas (6) and (7) could differentiate exosomes specific for CRC and CP patients. To test this hypothesis, the sequential application of the cutting filter and the circle masks Formulas (6) and (7) in a phasor plane were implemented for the whole dataset. The corresponding TPAF lifetime distribution functions are shown in Figure 8. Their parameters are presented in Table 1. 

**Figure 9:**
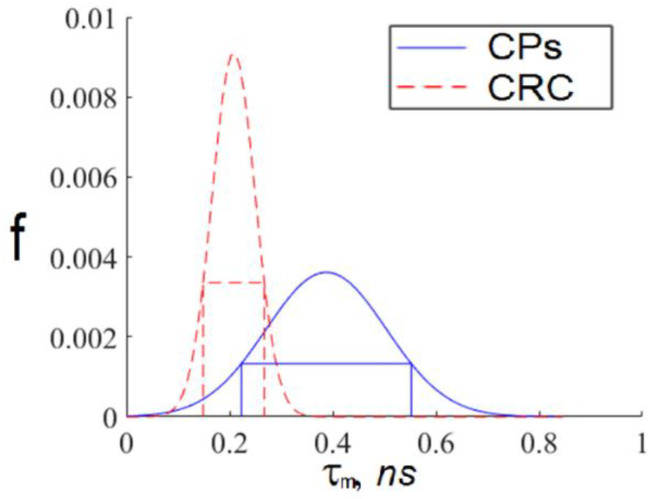
Interval approximations of the distribution functions of the short and long average TPAF lifetimes presented in Figure 8. These curves were calculated using Gaussian approximation of the data presented in Table 1. Notations on the legend: CPs—the exosome samples from the whole CRC patient group; CRC—the exosome samples from the whole CP patient group.

We constructed interval estimates for the distributions described by the parameters presented in Table 1, and the results are shown in Figure 9.

Using these interval approximations, we could construct a CRS diagnostics criterion as follows:

1. For every exosome in an FLIM image, we calculated the TPAF average lifetime $t_{m}$. If the $t_{m}\;$value fell within the intersection of the intervals corresponding to short and long lifetime interval approximations, then this exosome was ignored. Otherwise, we assigned this exosome the appropriate index: “h”, corresponding to a CP-associated exosome, or “c”, corresponding to a CRC-associated exosome.
2. For a whole FLIM dataset corresponding to a definite participant, we calculated the ratio as follows:
  (8) $$N_{ch}=\frac{\sum N_{c}}{\sum N_{h}+\sum N_{c}},$$
  where $N_{ch}$ is the relative number of CRC-associated exosomes in the FLIM dataset, $N_{c}$ is the number of CRC-associated exosomes, and $\;N_{h}$ is the number of “non-cancerous” (CP-associated) exosomes. If the $N_{ch}$ value is large, it is a sign of possible CRC presence.

**Figure 10:**
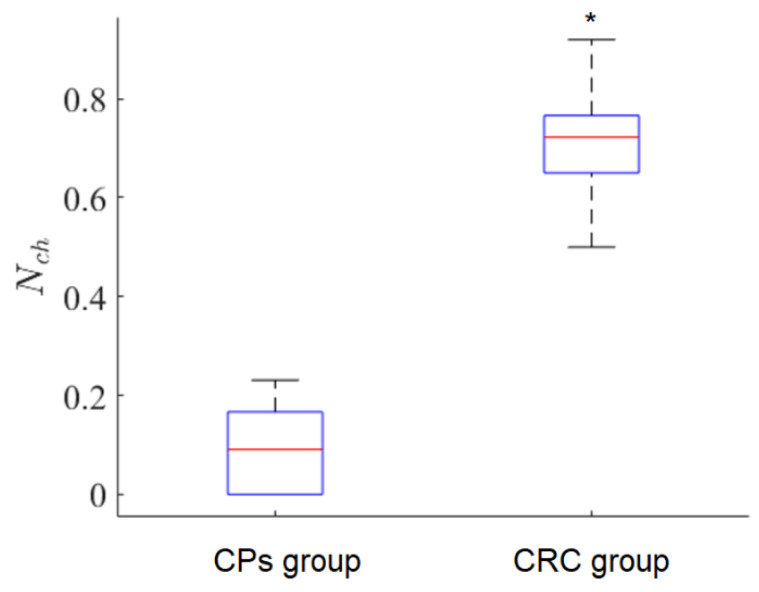
A box diagram for $N_{ch}\;$values for CP and CRC patient groups, * *p*-value is $3.35\cdot 10^{-5}.$ Here, $N_{ch}$ is the relative number of CRC-associated exosomes in the FLIM dataset calculated according to Formula (8).

The results of applying this criterion to CP and CRC patient groups are presented in Figure 10.

The relative number of CRC exosomes in the FLIM dataset was very small in the CP patient group, whereas the opposite situation was observed in the CRC patient group. In order to evaluate the statistical difference between the CP and CRC patient groups in terms of CP- and CRC-associated exosome content in blood plasma samples, the empirical value of the Mann–Whitney criterion $U_{c}$ was calculated [35]. Consider the hypothesis $H_{0}$ about the insignificance of differences between groups. The hypothesis is accepted if the critical value $U_{c}$ is less than the empirical $U_{e}$. In our case, for the probability α=0.01, the critical value was $U_{c}=34$ and the empirical value was $U_{e}=1$, i.e., the $H_{0}\;$hypothesis was not accepted.

## 4. Conclusions

Two classes of exosomes differentiated by average TPAF lifetime $t_{m}\;$were discovered in blood plasma samples from CP and CRC patients. Exosomes with short TPAF lifetimes were practically absent in blood plasma samples from the CP patients, whereas the opposite situation was the case for exosomes with long TPAF lifetimes. The TPAF average lifetime $t_{m}$ was shown to be (0.2445 ± 0.0054) ns for CRC-associated exosomes and (0.4071 ± 0.0114) ns for CP-associated exosomes.

Therefore, the CRS diagnostics criterion can be implemented as follows. For an exosome sample from a studied participant, the TPAF average lifetime $t_{m}$ can be measured and the relative number of CRC-associated exosomes in the FLIM dataset $N_{ch}\;$can be calculated considering the $t_{m}$ intervals for CRC-associated exosomes and “non-cancerous” (CP-associated) exosomes mentioned above. According to the data presented in Figure 10, if $N_{ch}$ > 0.5, the probability of CRC is high, and if $N_{ch}$ < 0.3, the probability of CRC is low. As $N_{ch}$ changes continuously in the interval [0, 1], collecting larger datasets potentially allows one to construct a scale of CRC severity in terms of $N_{ch}$ values. The nature of the suggested criterion based on peculiarities of cancer-associated exosomes’ chemical cargo, reflected in FLIM data, needs aditional research.

## Figures and Tables

**Table 1:** Mean and standard deviations for the distributions shown in Figure 8.

	Mean Value, ns	Standard Deviation, ns
The short average TPAF lifetime distribution	0.21	0.06
The long average TPAF lifetime distribution	0.43	0.19

## Data Availability

The data presented in this study are available on request from the corresponding author. The data are not publicly available due to privacy or ethical restrictions.
